# How To Publish: Preparing Manuscripts for Clarity, Transparency, Scholarly Integrity, and Success

**DOI:** 10.1093/geroni/igaa057.3159

**Published:** 2020-12-16

**Authors:** Suzanne Meeks

## Abstract

The GSA publications team sponsors this annual symposium to assist prospective authors to successfully publish their gerontological scholarship in GSA’s high impact and influential journals. The first part of the session will include five brief presentations from the Editors-in-chief of Journals of Gerontology-Series B, Social and Psychological Sciences, The Gerontologist, and Innovation in Aging, plus one of GSA’s managing editors. We will integrate practical tips with principles of publication ethics and scholarly integrity. The topics will be as follows: (1) preparing your manuscript, including how to choose the right journal; (2) strong and ethical scholarly writing for multidisciplinary audiences; (3) transparency, documentation, and Open Science; (4) successfully responding to reviews; and (5) working with Scholar One. Following these presentations, we will hold round table discussions with editors from the GSA journals portfolio. At these roundtables, editors will answer questions related to the podium presentations and other questions specific to each journal. Intended audiences include emerging and international scholars, and authors interested in learning more about best practices and tips for getting their scholarly work published.

